# Serum Cytokine Profiles Differentiating Hemorrhagic Fever with Renal Syndrome and Hantavirus Pulmonary Syndrome

**DOI:** 10.3389/fimmu.2017.00567

**Published:** 2017-05-18

**Authors:** Svetlana F. Khaiboullina, Silvana Levis, Sergey P. Morzunov, Ekaterina V. Martynova, Vladimir A. Anokhin, Oleg A. Gusev, Stephen C. St Jeor, Vincent C. Lombardi, Albert A. Rizvanov

**Affiliations:** ^1^Nevada Center for Biomedical Research, Reno, NV, USA; ^2^Institute of Fundamental Medicine and Biology, Kazan Federal University, Kazan, Russia; ^3^Department of Microbiology and Immunology, University of Nevada School of Medicine, Reno, NV, USA; ^4^Instituto Nacional de Enfermedades Virales Humanas “Dr. Julio I. Maiztegui”, Pergamino, Argentina; ^5^Department of Pathology, University of Nevada School of Medicine, Reno, NV, USA; ^6^Kazan State Medical University, Kazan, Russia; ^7^Preventive Medicine and Diagnosis Innovation Program, Center for Life Science Technologies, Yokohama, Japan

**Keywords:** hemorrhagic fever with renal syndrome, hantavirus pulmonary syndrome, nephropathia epidemica, cytokine profile, blood serum

## Abstract

Hantavirus infection is an acute zoonosis that clinically manifests in two primary forms, hemorrhagic fever with renal syndrome (HFRS) and hantavirus pulmonary syndrome (HPS). HFRS is endemic in Europe and Russia, where the mild form of the disease is prevalent in the Tatarstan region. HPS is endemic in Argentina, as well as other countries of North and South American. HFRS and HPS are usually acquired via the upper respiratory tract by inhalation of virus-contaminated aerosol. Although the pathogenesis of HFRS and HPS remains largely unknown, postmortem tissue studies have identified endothelial cells as the primary target of infection. Importantly, cell damage due to virus replication, or subsequent tissue repair, has not been documented. Since no single factor has been identified that explains the complexity of HFRS or HPS pathogenesis, it has been suggested that a cytokine storm may play a crucial role in the manifestation of both diseases. In order to identify potential serological markers that distinguish HFRS and HPS, serum samples collected during early and late phases of the disease were analyzed for 48 analytes using multiplex magnetic bead-based assays. Overall, serum cytokine profiles associated with HPS revealed a more pro-inflammatory milieu as compared to HFRS. Furthermore, HPS was strictly characterized by the upregulation of cytokine levels, in contrast to HFRS where cases were distinguished by a dichotomy in serum cytokine levels. The severe form of hantavirus zoonosis, HPS, was characterized by the upregulation of a higher number of cytokines than HFRS (40 vs 21). In general, our analysis indicates that, although HPS and HFRS share many characteristic features, there are distinct cytokine profiles for these diseases. These profiles suggest a strong activation of an innate immune and inflammatory responses are associated with HPS, relative to HFRS, as well as a robust activation of Th1-type immune responses. Finally, the results of our analysis suggest that serum cytokines profiles of HPS and HFRS cases are consistent with the presence of extracellular matrix degradation, increased mononuclear leukocyte proliferation, and transendothelial migration.

## Introduction

Hantaviruses are negative strand RNA viruses carried by rodents, insectivores, and bats. The epidemiology of hantaviruses reflects the distribution of their primary rodent hosts (1) and can be divided into two groups based on clinical manifestation in human: hantavirus pulmonary syndrome (HPS) and hemorrhagic fever with renal syndrome (HFRS). HPS is endemic in America and diagnosed in many countries including Argentina and USA (2, 3). Several hantaviruses, including Andes and Sin Nombre, were linked to HPS (1). In contrast, HFRS is exclusively diagnosed in Europe and Asia (1, 4, 5). In Europe, a mild form of HFRS, also referred as nephropathia epidemic (NE), is clinically distinguished (6, 7). HFRS/NE is diagnosed in many countries in Europe, including Western part of Russia, where endemically active regions include the republic of Tatartsan (8). Puumala virus (PUUV) is primarily identified as the causative agent of HFRS/NE in Tatarstan (9).

Hantaviruses cause asymptomatic infection in their rodent hosts, whereas in human, infection with Andes, Sin Nombre, and Puumala can result in an acute and sometimes fatal disease. It is believed that humans acquire infection by inhaling virus-contaminated aerosols (10). As a general rule, humans can only become infected after direct contact with infected rodents or their excreta; however, there has been documented cases of Andes virus being spread from person-to-person (11, 12).

Hantavirus pulmonary syndrome is an acute severe disease characterized by pneumonia, cardiovascular failure, and shock (13). As the respiratory symptoms worsen, the disease progresses into the cardiopulmonary phase characterized by respiratory distress. In a few hours, patients become hypotensive and develop tachycardia, which can lead to cardiovascular shock (13). Additionally, some patients will present with hemorrhagic manifestations (14, 15). The convalescent phase can last for months, especially in patients requiring mechanical ventilation. It has been suggested that the rates of HPS fatalities vary with the hantavirus strain. For example, Figueiredo et al. reported that the hantavirus strain Araraquara is associated with a more severe presentation of HPS as compared to the Juquitiba strain (15).

Clinically, HFRS manifests with bleeding disorders and kidney dysfunction (16–18). Later, hemorrhages appear, which vary from small petechia to severe internal bleeding (16, 17). In some severe cases, disseminated intravascular coagulation syndrome can develop, which is considered as one of the primary causes of death in HFRS (19). Kidney pathology is present in all cases, progressing through several stages of kidney dysfunction including oliguria, polyuria, and convalescent (18, 20).

*In vitro* studies and postmortem observations have shown that hantaviruses are not cytopathic (21–24). Therefore, HPS and HFRS pathogenesis cannot be explained by direct tissue damage due to viral replication. Reactions of the organism to hantavirus infection, particularly the control of inflammatory cytokine expression, have been suggested as a key factor in disease pathogenesis. In accordance, an increased tissue infiltration with mononuclear leukocytes is a hallmark of HPS and HFRS (23–25). It has been suggested that the leukocyte infiltration, found in postmortem HPS and HFRS tissues, may be the result of an increased expression of inflammatory cytokines (24, 26). Additionally, high serum levels of inflammatory cytokines have been described for both HPS and HFRS (27–29). Furthermore, it appears that the severity of the disease associates with pro-inflammatory cytokine expression in patients with hantavirus infection. For example, Saksida et al. demonstrated that the level of serum pro-inflammatory cytokines was higher in HFRS patients infected with Dobrava virus as compared to PUUV-infected patients (30). Additionally, our previous report points out the potential role of Th1-type immune response in the severity of NE (28). PUUV infection presents with a milder form of the disease and lower mortality rate when compared to Dobrava virus infections. Therefore, it could be suggested that inflammatory cytokine expression may determine the severity of clinical presentations and, potentially impact the mortality rates (6, 31).

In the present study, our analyses revealed that distinct cytokine profiles associated with HPS and HFRS. The HPS profile was suggestive of severe inflammatory responses when compared to that of HFRS. Additionally, our data demonstrate that HPS is characterized by the upregulation of Th1-type immune response early during infection. Pronounced upregulation of cytokines that facilitate innate immune response, especially natural killer (NK) cell function, was also observed in HPS cases relative to HFRS. Furthermore, activation of a mixed population of immune effector cells, including mononuclear and segmented leukocyte, is predicted in HPS cases based on the cytokine profile. Finally, the observed HPS and HFRS serum cytokine profiles are consistent with disease pathology characterized by increased mononuclear leukocyte proliferation, transendothelial migration, and degradation of extracellular matrix.

## Materials and Methods

### Subjects

One set of samples consisted of sera collected in Argentina during HPS outbreaks in Buenos Aires, Santa Fé, Entre Ríos, and Jujuy provinces. Of the 40 HPS serum samples from 30 total subjects (34 ± 3.5; 26 male, 4 female), 7 individual samples were from fatal cases, 29 were collected from those who survived, and no information was available with respect to the outcome of the other 4. Out of the 29 samples from survivors, serum samples from three cases were collected once at the early stage of the disease, and two sets of serum samples were collected from 13 patients, once during the early stage and once at a late stage of the disease.

Sixty-seven HFRS serum samples (38 ± 4.6; 52 male, 15 female) were collected from patients admitted into Republican Clinical Hospital for Infectious Disease, Republic of Tatarstan. Serum from 33 HFRS cases was collected twice (early and convalescent), while a single serum sample was obtained from one patient. Diagnoses of HFRS and HPS were based on clinical presentation and were serologically confirmed by detection of anti-hantavirus antibodies. In some cases, the diagnosis was confirmed by PCR. Serum samples from 55 healthy individuals (37 ± 5.1; 32 males, 13 females) matched by age, gender, and region were collected and served as controls. Samples collected in the Russian Federation were done so under a protocol approved by the Institutional Review Board of the Kazan Federal University and informed consent was obtained from each respective subject according to the guidelines approved under this protocol (Article 20, Federal Law “Protection of Health Right of Citizens of Russian Federation” N323-FZ, 11.21.2011). Sample collection in Argentina was made under a protocol approved by the Institutional Review Board of the Instituto Nacional de Enfermedades Virales Humanas Argentina.

### Multiplex Analysis

Serum cytokine levels were analyzed using multiplex magnetic bead-based antibody detection kits following the manufacturer’s instructions (Bio-Rad, Hercules, CA, USA). In order to survey 48 individual analytes, the Bio-Plex Pro Human Cytokine 27-plex and 21-plex immunoassay kits were used. For each subject, 50 µl of serum was analyzed on a Luminex 200 analyzer (Luminex, Austin, TX, USA) utilizing MasterPlex CT control software and MasterPlex QT analysis software (MiraBio, San Bruno, CA, USA). The median fluorescence intensities were determined using a minimum of 50 beads per analyte and serum concentrations were calculated using standard curves for each analyte generated with standards provided with the Bio-Plex kit. Each serum sample was analyzed in duplicates.

### Cytokine Network and Functional Analysis

Ingenuity Pathway Analysis (IPA) Software and DAVID 6.7 Software (32) packages were used independently, to examine cytokine-based enrichment of molecular pathways. The list of genes coding significantly altered cytokines (with threshold of ±2-fold in the average value of the cytokine level, *P* value < 0.05) were used and the pathways showing the enrichments with significance of *P* < 0.05 were identified and analyzed. Both platforms provided corroborative results and the same significantly enriched pathways lists.

### FANTOM5 Analysis

Raw cytokine levels, measured using the Bio-Plex system, were first normalized against the median level of the 48 cytokines in each sample. Per group median normalization was then carried out for two separate groups, Argentina (Andes) and Tartarstan (HFRS). For each cytokine/group, the level of a given cytokine was normalized to the median level across the group (including both controls and cases). In the Argentina and Tartarstan groups, there were also subgroups corresponding to sera taken upon admission to the hospital and sera taken upon discharge. The median fold changes were compared between cases and controls for each group or subgroup. Expression profiles of the upregulated cytokines were manually examined in the FANTOM5 datasets (PMID: 24670764, PMID: 25678556), to determine what primary cell types they are expressed by and whether they are induced upon pathogenic stimuli.

### Statistical Analysis

Statistical analysis was performed using Statistica and XLSTAT software (StatSoft, Tulsa, OK, USA, and Addinsoft, New York, NY, USA, respectively). Statistical analysis was performed using Student’s *t*-test for comparisons between individual experimental groups. Data are presented as mean < SE. Significance was established at a value of *P* < 0.05.

## Results

### Serum Samples

Hantavirus pulmonary syndrome and NE serum samples were collected at different time points during hospitalization. Due to the nature of the disease, two-time point collection was not always feasible. For example, only a single serum sample was collected from each of the seven fatal HPS cases. The majority of HPS samples (12 samples) from Argentina were collected upon admission to the hospital, while a small number of samples (seven samples) were also obtained before discharge. NE and HPS sera were obtained as clinical surplus material; therefore, no patient identifiers were available to match the first and second samples. Information provided was limited to diagnosis and virus strain. HPS sera from Argentina were mostly Lechiguanas strain, with the exception of three samples confirmed by PCR to be the Orán strain. HFRS samples were collected in the Republic of Tatarstan, an area identified as endemic for Puumala hantavirus (8). Based on these data, we partitioned the serum samples into Andes and PUUV infections.

### Serum Cytokine Profile

#### HPS Cases from Argentina

Two sets of HPS serum samples were collected, early at the time of hospitalization (7.05 ± 0.98 days) and during the convalescent period (42.2 ± 6.9 days). Serum levels of 41 cytokines were upregulated during the early stage of the disease (Table 1). The majority of these cytokines, a total of 35, remained upregulated at the late stage disease. For most of the cytokines, serum concentration had declined by the late stage, while levels of some cytokines remained upregulated (CXCL1, LIF, CXCL9, TNFβ) or was further elevated (CXCL10, CCL2, IL-1α, IL-2RA, IL-3, IL-12p40, CCL27, CCL7, bNGF, SCF, TRAIL). When early- and late-stage serum cytokine levels were compared, the only significant differences detected were in the concentrations of CXCL10 and MCSF. Interestingly, higher CXCL10 and lower MCSF levels were found in the late stage serum as compared to that in early time points of the disease.

**Table 1:** **Serum cytokine levels in early and late hantavirus pulmonary syndrome (HPS)**.

Analyte	Control (*n* = 10; pg/ml)	HPS early (*n* = 16; pg/ml)	HPS late (*n* = 13; pg/ml)	P*	P*	*P*^#^
IL-1α	86.7 ± 9.1	393.3 ± 10.7	411.1 ± 18.9	0.0001	0.0001	
IL-1RA	327.4 ± 62.5	1,134.0 ± 408.1	657.4 ± 210.6	0.002		
IL-2^a^	17.5 ± 1.7	88.5 ± 44.5	20.6 ± 1.9	0.005		
IL-2RA^a^	138.7 ± 12.2	1,375.0 ± 122.5	1,426.9 ± 489.9	0.0001	0.0001	
IL-3	82.1 ± 11.7	2,444.9 ± 215.4	2,595.9 ± 661.4	0.0001	0.0001	
IL-4^a^	8.8 ± 8.8	21.9 ± 5.1	12.7 ± 1.2	0.0001	0.01	
IL-7^a^	67.9 ± 4.9	130.0 ± 31.2	94.9 ± 3.5	0.002	0.02	
IL-9	25.8 ± 3.1	85.4 ± 20.5	56.7 ± 12.3	0.0001	0.0008	
IL-10	45.2 ± 4.9	186.8 ± 101.8	101.8 ± 15.8	0.01	0.0001	
IL-12p40	159.7 ± 23.5	4,295.9 ± 513.1	4,732.8 ± 1,324.4	0.0001	0.0001	
IL-12p75	44.6 ± 4.6	100.0 ± 14.7	79.8 ± 6.4	0.0001	0.002	
IL-13	52.4 ± 4.0	97.4 ± 21.1	77.3 ± 2.6	0.001	0.01	
IL-15	33.2 ± 2.7	68.2 ± 14.1	51.3 ± 3.2	0.0003	0.007	
IL-16	169.3 ± 35.8	2,759.3 ± 295.2	2,586.8 ± 625.5	0.0001	0.0001	
IL-17A^a^	84.7 ± 15.7	208.9 ± 30.9	172.3 ± 21.7	0.0003	0.02	
IL-18	444.4 ± 71.3	854 ± 60.2	784 ± 143	0.003		
CCL2	173.4 ± 17.0	265.2 ± 34.7	460.8 ± 111.7	0.01	0.0001	
CCL4	136.6 ± 14.4	381.3 ± 138.9	212.9 ± 26.8	0.003	0.03	
CCL7	128.9 ± 22.6	455.3 ± 71.4	579.6 ± 221.9	0.0001	0.0001	
CCL11^a^	85.9 ± 8.9	202.0 ± 62.2	140.3 ± 26	0.002	0.02	
CCL27^a^	836.3 ± 233.7	1,550.0 ± 76.6	1,664.2 ± 82.5	0.005	0.01	
CXCL1	404.8 ± 69.4	1,181.5 ± 100.4	1,025.5 ± 70.4	0.0001	0.0003	
CXCL9	734.8 ± 140.5	1,949.4 ± 136.5	1,911.5 ± 368.2	0.0001	0.002	
CXCL10	415.3 ± 77.5	2,383.9 ± 600.8	5,188.1 ± 981.2	0.0001	0.0001	0.013
CXCL12	213.7 ± 23.9	3,577.9 ± 116.3	3,404.8 ± 145.6	0.0001	0.0001	
bFGF^a^	42.3 ± 5.4	108.5 ± 27.9	65.9 ± 5.8	0.0005		
bNGF	79.9 ± 10.3	278.3 ± 16.6	305.9 ± 42.1	0.0001	0.0001	
GMCSF	42.9 ± 5.9	174.6 ± 68.9	82.5 ± 13	0.001	0.01	
HGF	705.6 ± 134.5	3,059.5 ± 333.7	2,319.3 ± 434.5	0.0001	0.0001	
IFNα	71.0 ± 7.3	466.9 ± 30.7	387.4 ± 85.7	0.0001	0.0001	
IFNγ	69 ± 11.9	190.1 ± 41.9	171.5 ± 19.2	0.0003	0.0008	
LIF	177.7 ± 24.1	742.5 ± 57.2	708.5 ± 109.1	0.0001	0.0001	
MCSF	206.3 ± 30.9	876.9 ± 59.5	607.3 ± 106.2	0.0001	0.0001	0.02
MIF	229.5 ± 59.1	1,781.1 ± 308.5	1,072.1 ± 111.9	0.0001	0.0001	
PDGF-BB^a^	1,759.8 ± 124.5	2,568.6 ± 307.3	2,057.7 ± 316.9	0.005		
SCF	268.3 ± 39.9	1,308.1 ± 78.8	1,436.9 ± 268.9	0.0001	0.0001	
SCGFb	2,037.4 ± 370.3	37,608.9 ± 1796.4	31,720.5 ± 2857.5	0.0001	0.0001	
TNFα^a^	84.4 ± 10.3	234.9 ± 42.9	199.5 ± 19.9	0.0001	0.0001	
TNFβ	84.3 ± 11.0	264.1 ± 20.7	272.4 ± 54.3	0.0001	0.0001	
TRAIL-	157.6 ± 18.4	1,655.4 ± 178.3	1,840.2 ± 654.5	0.0001	0.0001	
VEGF^a^	140.5 ± 12.7	248.4 ± 36.2	173.0 ± 28.2	0.0006		

*P-values for controls vs early HPS cases.

**P-values for controls vs late HPS cases.

*^#^P-values for late vs early HPS cases*.

*^a^Values differ significantly only in early serum from non-fatal HPS cases and not in fatal cases*.

Serum samples from seven fatal HPS cases were collected upon admission to the hospital. The average date of serum collection from fatal cases was 6.1 ± 1.18, which is close to those collected in early phase non-fatal cases (7.05 ± 0.98 days). Therefore, early phase of non-fatal cases and fatal cases could be compared (Table 2). Serum levels of 30 cytokines were upregulated in fatal HPS cases. Although the majority of these cytokines overlapped with those upregulated in non-fatal cases, there were cytokines uniquely elevated in fatal cases. For example, IL-2, IL-2RA, IL-4, IL-7, IL-17A, CCL4, CCL11, CCL27, bFGF, PDGF-BB, TNFα, and VEGF (Table 1) were upregulated in non-fatal cases, while IL-6 was significantly upregulated only in fatal cases when compared to healthy controls (Table 2). When cytokines upregulated in fatal and non-fatal cases were compared, significant differences in serum levels of IL-2RA, IL-18, CXCL1, HGF, MCSF, MIF, CXCL9, and SCF were identified (Table 2).

**Table 2:** **Serum cytokine levels in survived and fatal hantavirus pulmonary syndrome (HPS) cases**.

Analyte	Control (*n* = 10; pg/ml)	Survived (*n* = 29; pg/ml)	Fatal (*n* = 7; pg/ml)	P*	P**
IL-1α	86.7 ± 9.1	393.3 ± 10.7	404.2 ± 15.9	0.0001	
IL-2RA	138.7 ± 12.2	1,375 ± 122.6	1,903.6 ± 240.5	0.0001	0.04
IL-3	82.1 ± 11.7	2,444.9 ± 215.4	2,813.8 ± 287.4	0.0001	
IL-6^a^	74.2 ± 18.7	137.7 ± 54.4	422.9 ± 367.4	0.01	
IL-9	25.8 ± 3.1	85.4 ± 20.5	94.9 ± 55.1	0.001	
IL-10	45.2 ± 4.9	186.8 ± 101.8	92.8 ± 17.9	0.0026	
IL-12p40	159.7 ± 23.5	4,295.9 ± 513.1	5,776.9 ± 1,102.2	0.0001	
IL-12p75	44.6 ± 4.6	100 ± 14.7	73.6 ± 9.5	0.03	
IL-13	52.4 ± 4.0	97.4 ± 21.0	76.3 ± 4.8	0.04	
IL-15	33.2 ± 2.7	68.2 ± 15.0	51.5 ± 5.5	0.02	
IL-16	169.3 ± 35.7	2,759.3 ± 295.2	3,848.4 ± 696.9	0.0001	
IL-18	444.4 ± 71.3	854.2 ± 60.3	1,640.7 ± 532.9	0.0001	0.03
CCL2	173.4 ± 17.0	256.2 ± 34.7	313.4 ± 104.5	0.02	
CCL7	128.9 ± 22.6	455.3 ± 71.4	550.4 ± 139.7	0.0001	
CXCL1	404.9 ± 69.3	1,181.5 ± 100.4	2,283.3 ± 734.4	0.0001	0.03
CXCL9	734.8 ± 140.5	1,949.5 ± 136.5	2,822.3 ± 306.1	0.0001	0.006
CXCL10	415.3 ± 77.5	2,383.9 ± 600.8	3,175.6 ± 1,400.3	0.0001	
CXCL12	213.7 ± 23.9	3,577.9 ± 116.3	3,865.8 ± 145.5	0.0001	
bNGF	79.9 ± 10.3	278.3 ± 16.7	258.6 ± 5.3	0.0001	
GMCFS	42.9 ± 5.9	174.6 ± 68.9	82.8 ± 19.4	0.03	
HGF	705.6 ± 134.5	3,059.5 ± 333.7	7,513.2 ± 1,896.8	0.0001	0.002
IFNα	71.0 ± 7.3	466.9 ± 30.7	528.6 ± 23.7	0.0001	
IFNγ	69.0 ± 11.9	190.1 ± 41.9	175.3 ± 27.9	0.003	
LIF	177.7 ± 24.0	742.5 ± 57.2	838.4 ± 63.7	0.0001	
MCSF	206.3 ± 30.9	876.9 ± 59.5	1,365.4 ± 210.9	0.0001	0.006
MIF	299.5 ± 59.1	1,781.1 ± 308.5	4,087.6 ± 1,121.5	0.0001	0.01
SCF	268.3 ± 39.9	1,308.1 ± 78.8	1,697 ± 214.2	0.0001	0.04
SCGFb	2,037.4 ± 370.3	37,609 ± 1,796.5	38,632.7 ± 1,168.9	0.0001	
TNFβ	84.3 ± 11.0	264.1 ± 20.7	282.9 ± 36.1	0.0001	
TRAIL	157.6 ± 18.4	1,655.4 ± 178.3	1,954.9 ± 216.2	0.0001	

*P-values for fatal HPS cases vs controls.

**P-values for fatal vs non-fatal HPS cases.

*^a^Values differ significantly only in fatal HPS cases and not in early non-fatal cases*.

#### HFRS Cases from Russia

Serum cytokines of HFRS cases can be divided into two groups, with 22 cytokines being upregulated and 12 cytokines downregulated (Table 3). Sixteen cytokines were significantly upregulated in the early stage of HFRS, while 11 were significantly downregulated as compared to controls. At the late stage of the disease, 18 cytokines were upregulated and 12 downregulated. Those that were downregulated during the convalescent phase differed from those in the early stage. For example, compared to controls, the level of CCL5 changed significantly only during the late stage of the disease, while the serum level of bFGF was significantly downregulated only during the early stage of the disease. When upregulated cytokines were compared, nine cytokines (IL-1β, IL-1RA, IL-2, IL-4, IL-6, IL-7, IL-12p75, IFNγ, and TNFα) differed significantly between the early and late stage of the disease. Interestingly, the average serum concentrations of these cytokines were higher in the late stage as compared to the early stage of the disease.

**Table 3:** **Serum cytokine levels in early and late hemorrhagic fever with renal syndrome (HFRS) cases**.

Analyte	Control (*n* = 45)	HFRS early (*n* = 34)	HFRS late (*n* = 33)	P*	P**	*P*^#^
**Upregulated cytokines**						
IL-1β	32.04 ± 4.7	52.5 ± 5.9	96.2 ± 19.6	0.007	0.0001	0.03
IL-1RA	327.4 ± 62.4	330.4 ± 18.5	589.8 ± 118.5		0.03	0.03
IL-2	17.5 ± 1.7	30.9 ± 0.9	39.6 ± 3.8		0.0001	0.03
IL-3	82.1 ± 11.7	504.7 ± 53.8	464.3 ± 59.7	0.0001	0.0001	
IL-4	8.8 ± 0.6	10.9 ± 0.1	12.1 ± 0.5	0.0001	0.0003	0.04
IL-5	18.9 ± 1.7	40.9 ± 0.2	40.9 ± 0.2	0.0001	0.0001	
IL-6	74.2 ± 18.7	71.8 ± 4.5	102.8 ± 14.8			0.04
IL-7	67.9 ± 4.9	88.3 ± 0.2	91.0 ± 1.3	0.001	0.0004	0.04
IL-12p40	159.7 ± 23.6	915.6 ± 100.9	890.0 ± 105.9	0.0001	0.0001	
IL-12p75	44.6 ± 4.6	29.8 ± 1.7	43.4 ± 5.4	0.01		0.01
IL-13	52.4 ± 4.1	73.4 ± 0.2	75.0 ± 0.8	0.0001	0.0001	0.05
IL-16	169.3 ± 35.8	830.1 ± 134.3	544.1 ± 108.4	0.0001	0.0001	
CCL4	136.6 ± 14.4	227.7 ± 1.9	225.6 ± 32.7	0.0008	0.005	
CXCL9	734.8 ± 140.5	2,741.3 ± 1,228.9	1,293.9 ± 328.1	0.03		
CXCL10	415.3 ± 77.5	2,057.5 ± 405.1	1,802.4 ± 408.4	0.0001	0.0001	
CXCL12	213.7 ± 23.9	384.0 ± 5.9	383.5 ± 7.1	0.0001	0.0001	
GMCSF	42.9 ± 5.9	60.9 ± 7.4	77.9 ± 9.9		0.001	
IFNγ	69 ± 11.9	67.5 ± 16.2	488.2 ± 115.1		0.0001	0.0004
MIF	229.5 ± 59.1	4,615.1 ± 941.4	2,901.3 ± 740.9	0.0001	0.0001	
SCGFb	2,037.4 ± 370.3	11,926.7 ± 1,322.5	10,474.7 ± 1,372.6	0.0001	0.0001	
TNFα	84.4 ± 10.3	62.1 ± 1.2	101.9 ± 20.2			0.04
VEGF	140.5 ± 12.7	215.9 ± 18.2	251.5 ± 26.3	0.0006	0.0001	
**Downregulated cytokines**						
IL-1α	86.7 ± 9.1	30.5 ± 0.9	30.1 ± 1.2	0.0001	0.0001	
IL-2RA	138.7 ± 12.2	102.4 ± 6.7	99.8 ± 4.3	0.02	0.02	
IL-18	444.4 ± 71.3	114.4 ± 8.5	121.9 ± 12.2	0.0001	0.0007	
CCL5	6,348.9 ± 581.2	5,072.2 ± 265.2	4,768.8 ± 245.1		0.04	
CCL27	836.3 ± 133.7	335.8 ± 42.5	266.3 ± 34.1	0.0001	0.001	
CXCL1	404.8 ± 69.4	162.3 ± 17.8	154.1 ± 19.9	0.007	0.006	
bFGF	42.3 ± 5.4	15.9 ± 1.7	30.6 ± 5.7	0.0002		0.01
bNGF	79.9 ± 10.3	22.9 ± 0.5	22.9 ± 0.8	0.0001	0.0001	
LIF	177.7 ± 24.0	85.5 ± 2.6	85.5 ± 2.9	0.003	0.003	
MCSF	206.3 ± 30.9	117.5 ± 12.9	100.8 ± 10.6	0.02	0.01	
SCF	268.3 ± 39.9	164.7 ± 17.9	146.3 ± 16.4	0.05	0.02	
TNFβ	84.3 ± 18.4	35.1 ± 0.7	34.9 ± 0.7	0.0006	0.0007	

*P-values for controls vs early HFRS cases.

**P-values controls vs late HFRS cases.

*^#^P-values for early and late HFRS cases*.

#### HFRS vs HPS Cytokine Profile

While the serum levels of 12 cytokines were lower in early HFRS relative to the controls, no cytokines were observed to be downregulated in HPS cases (Tables 1 and 3, respectively). All 12 cytokines downregulated during the early stage of HFRS were upregulated in HPS cases.

Twenty-two cytokines were upregulated in the sera of early-stage HFRS cases, while 41 cytokines were upregulated in early HPS cases (Tables 1 and 3, respectively). Furthermore, only 14 cytokines were upregulated in both HFRS and HPS cases at the early stage (IL-1RA, IL-2, IL-4, IL-7, IL-12p40, IL-12p75, IL-13, IL-16, CXCL9, CXCL10, CXCL12, MIF, SCGFb, and VEGF). As the disease progressed, 17 cytokines were upregulated in HFRS cases, while a much larger group of cytokines, total of 35, was upregulated in HPS cases (Tables 1 and 3, respectively). Among these cytokines, 13 (IL-3, IL-4, IL-7, IL-12p40, IL-13, IL-16, CCL4, CXCL10, CXCL12, GMCSF, IFNγ, MIF, and SCGFb) were upregulated in the late stages of both HFRS and HPS. It should be noted that the profiles of overlapping cytokines differed in the late stages of HFRS and HPS as compared to that during the early phases of the disease. Only seven cytokines (IL-13, CXCL10, IL-12p40, IL-16, MIF, SCGFb, and CXCL12) were upregulated in early and late stages of HFRS and HPS, while levels of IL-12p75 and CXCL9 remained elevated only in late-stage HPS samples.

### FANTOM5 Analysis

FANTOM5 analysis identified CXCL12 as the most upregulated cytokine in the serum of HPS cases. CXCL12 is induced in response to strong pro-inflammatory stimuli such as TNFα and IL-1 (33). CXCL12 is produced by a plethora of cells including endothelial cells, smooth muscle cells, and macrophages; however, platelets are also a major source of this cytokine in circulation (34–38). It has been shown that CXCL12 increases angiogenesis by binding to heparan sulfate proteoglycans on endothelial cells (33). Therefore, it could be suggested that HPS cases are characterized by increased angiogenesis, possibly due to endothelial damage.

Hemorrhagic fever with renal syndrome cases were characterized by a strong expression of MIF, IL-12p40, IL-3, IL-16, CXCL9, CCL27, CXCL1, and HGF. On the contrary, upregulation of MIF, IL-12p40, IL-3, and IL-16 was modest in HPS, while activation of CXCL12 expression was the characteristic feature of HPS. This observation suggests that virus strain-based reactions to hantavirus infection contributes to the profile. Strong upregulation of IL-12p40, CXCL9, IL-16, and CCL27, mainly produced by activated mononuclear leukocytes including lymphocytes, monocytes, and dendritic cells, are suggestive of a role for mononuclear leukocytes in pathogenesis of HFRS.

### IPA Analysis of Serum Cytokine Profile in HFRS and HPS

Differentially expressed serum cytokines levels for HFRS and HPS cases, as well as fatal and non-fatal HPS cases, were further investigated by functional pathway analysis using two independent pathway enrichment analysis engines. First, the HFRS and HPS cytokine profiles of early samples were compared. The most important canonical pathways altered in the early phase of HFRS include cytokine–cytokine receptor interaction chemokine signaling, MAPK signaling, and neurotropin signaling pathways. For early HPS, a larger number of pathways were changed including cytokine–cytokine receptor interaction, RIG-I-like signaling, chemokine signaling, STAT signaling, cytosolic DNA-sensing, regulation of autophagy, TOLL-like receptor signaling, and NOD-like receptor signaling pathways. Additionally, pathways regulating antigen processing and presentation, as well as NK-cell-meditated cytotoxicity, were identified as significant in early-stage HPS cases.

Serum cytokine profile analysis of samples from late phase of HFRS did not reveal significantly altered pathways as compared to healthy controls. Analysis of serum samples from the late phase of HPS revealed fewer pathways as compared to that during the early phase of the disease and included NOD-like receptor signaling, cytokine–cytokine receptor interaction and RIG-I-like receptor signaling. When fatal and non-fatal HPS cases were compared, the cytokine–cytokine receptor interaction and JAK–STAT signaling pathways had significant differences. The serum cytokine profiles suggest that the JAK–STAT signaling pathway was suppressed in fatal cases as compared to non-fatal HPS cases.

## Discussion

Hemorrhagic fever with renal syndrome and HPS are acute infectious diseases caused by a distinct group of hantaviruses circulating among small rodents. In Tatarstan, HFRS is associated with infection by Arvicolinae-borne hantaviruses and HPS is a disease caused by Sigmodontinae-associated hantaviruses (39). Although clinically HPS and HFRS may overlap (40–42), pneumonia and cardiovascular insufficiency are central to HPS, while renal involvement and dysregulation in blood coagulation being the main sequelae in HFRS. The mortality rates also differ, with HPS having a higher mortality rate than HFRS, 39.3 vs 0.4, respectively (6, 43). Although there are distinct clinical presentations associated with Sigmadontinae- and Arvicolinae-born hantaviruses, these viruses are non-cytopathic *in vitro* (21, 22). Furthermore, no virus replication-associated cell death has been reported in tissues collected postmortem from HFRS and HPS cases (23, 24), suggesting that a response to infection plays a major role in the pathogenesis of these diseases. Differences in serum cytokine expression are one of the most consistent findings in HPS and HFRS. Many studies have documented a significant upregulation of pro-inflammatory cytokines, leading to the “cytokine storm” hypothesis to explain the pathogenesis of hantavirus-caused diseases.

Our data provide evidence to support the role of cytokines in HPS and HFRS pathogenesis. Striking differences in cytokine profiles were found between HPS and HFRS samples. HPS, the more severe form of hantavirus zoonosis, is characterized by upregulated serum cytokine levels, with no cytokines being downregulated (Tables 1 and 3). On the contrary, HFRS cases were characterized by dichotomy in the serum cytokine profile (Table 3), with cytokines being both upregulated and downregulated. Additionally, we observed higher number of cytokines upregulated in HPS as compared to HFRS, 41 vs 22, respectively. These data suggest that the severity of the disease could be associated with a “cytokine storm” as the body’s response to infection.

Our data support previous observations that chemokines promoting mononuclear leukocyte migration are upregulated in hantavirus-infected patients (28). In this study, upregulation of CXCL9 and CXCL10 were characteristic for both HPS and HFRS throughout the course of the disease. Here, we extend the knowledge of chemokine expression in HPS and HFRS. For example, increased serum levels of IL-16, a chemoattractant acting on activated T lymphocytes, was found in both HPS and HFRS (44). Additionally, we have shown that CXCL12, a potent chemoattractant for mononuclear leukocytes, is upregulated in patients with hantavirus infection (45, 46). Furthermore, there were chemokines uniquely upregulated in HPS cases including CCL2, CXCL1, and CCL7. These cytokines are known to promote tissue migration of monocytes, memory T cells, and dendritic cells (47, 48).

CXCL1, a neutrophil chemoattractant produced by activated macrophages and epithelial cells (49, 50), was upregulated in HPS cases, while being downregulated in HFRS samples. This suggests that although leukocyte tissue infiltration is characteristic to both HPS and HFRS, there is a mixed phenotype of migrating immune effector cells in HPS cases including neutrophils and mononuclear leukocytes. Since neutrophil migration is often associated with tissue damage and vascular leakage, the role of neutrophil migration in HPS pathogenesis requires further investigation.

Early activation of leukocytes is evident in the serum of HPS cases and differs from HFRS. For example, IL-15 is upregulated only in HPS, suggesting activation and proliferation of T cells and NK cells. IL-15 functions through JAK kinase, STAT3, STAT5, and STAT6, which is supported by IPA analysis. Additionally, activation of GCSF was detected only in HPS cases, at both early and late stages of the disease, suggesting sustained proliferation, differentiation, and survival of monocytes and macrophages in HPS. Elevated serum levels of IL-3, SCGFb, and SCF in HPS indicates increased proliferation of bone marrow progenitors, and upregulation of M-CSF and GM-CSF further suggests that myeloid progenitor proliferation is increased in HPS. Also, it should be noted that levels of IFN-γ remained persistently increased in HPS cases throughout the course of the disease, whereas IFN-γ levels were upregulated during late -phase HFRS, consistent with our previous observations (28). IFN-γ is associated with activation of Th1-type immune responses and is produced by activated T helper cells, cytotoxic T lymphocytes (CTL), and NK cells.

Our findings that IL-18 was upregulated in the serum of HPS cases, while being downregulated in the serum from those with HFRS, further corroborate the activation of Th1. IL-18 is a strong activator of Th1-type immunity, and alone or together with IL-12, upregulates the production of IFNγ by T cells and NK cells (51). These data suggest that early activation of NK cells and CTL is characteristic to HPS, while being less pronounced in HFRS. Excessive activation of NK cells and CTL can cause tissue damage, which has been shown to play a role in the pathogenesis of organ damage (52, 53). Recently, Prescott et al. suggested that an adaptive immune response has no influence on hantavirus replication or disease pathogenesis, based on studies using a hamster model of HPS (54). However, clinical studies indicate the potential role of innate and adaptive immune responses in disease pathogenesis (55–58). Our data support the role of adaptive immune responses in the pathogenesis of hantavirus infection. Also, it could be suggested that early strong activation of Th1-type immune response may be associated with severe clinical presentations.

A strong inflammatory response is evident based on serum cytokine profiles for HFRS and HPS. For instance, upregulation of pro-inflammatory cytokines such as IL-1β, IL-6, TNFα, and MIF were found inHFRS and HPS cases. Although the elevation of IL-1β, IL-6, and TNFα in hantavirus cases is well documented, upregulation of serum MIF has not been described previously for either HPS or HFRS cases. MIF is a multifactorial cytokine with a strong pro-inflammatory activity, suggesting a role for MIF in increasing vascular permeability and tissue migration of immune effector cells. Furthermore, increased serum VEGF levels in HFRS and HPS cases also suggest that this pro-inflammatory activity can be promoted by MIF-induced upregulation of metalloproteases, resulting in degradation of the extracellular matrix (59–61). Additionally, MIF can facilitate leukocyte transendothelial migration by upregulation of the adhesion molecules VCAM-1 and ICAM-1 on endothelial cells and monocytes (62, 63). Although HFRS and HPS are characterized by an increased pro-inflammatory serum profile, as described above, the upregulation of IL-18 was unique to HPS. IL-18 has been classified as a cytokine promoting severe inflammatory reactions (64–66). Therefore, we suggest that the clinical presentation of HPS may be due, in part, to the high serum levels of IL-18. It has been demonstrated that IL-18 activation is regulated through the NOD-like receptor regulated pathway (67, 68). IPA analysis of serum cytokine profiles revealed NOD-like pathway involvement only in HPS cases, further suggesting a role for this pathway in HPS pathogenesis.

Our analysis of serum cytokine profiles in HPS and HFRS cases suggests that although HPS and HFRS share many features, there are distinct cytokine profiles differentiating these diseases. They include (1) a severe inflammatory response in HPS cases, where IL-18 may play a central role, (2) a robust and early activation of Th1-type immune response in HPS cases as compared to HFRS, and (3) a strong activation of an innate immune response, especially NK cells in HPS cases. The cytokine profiles are suggestive of degradation of the extracellular matrix, increased mononuclear leukocyte proliferation, and transendothelial migration in both HPS and HFRS.

## Ethics Statement

Samples collected in the Russian Federation were done so under a protocol approved by the Institutional Review Board of the Kazan Federal University and informed consent was obtained from each respective subject according to the guidelines approved under this protocol (Article 20, Federal Law “Protection of Health Right of Citizens of Russian Federation” N323-FZ, 11.21.2011). Sample collection in Argentina was made under a protocol approved by the Institutional Review Board of the Instituto Nacional de Enfermedades Virales Humanas Argentina.

## Author Contributions

SK: collecting data, data analysis, and writing manuscript. SL: HPS serum sample preparation, Argentina site. SM: HPS serum sample preparation, USA site. EM: HFRS samples preparation and collecting data, Russia site. VA: clinical data analysis and contribution into the discussion. OG: IPA analysis and contribution into discussion. SJ: manuscript editing and study team coordinator. VL: manuscript editing and contribution into discussion. AR: project management and coordination between Argentina, USA, Russia, and Japan sites and intellectual contribution into discussion.

## Conflict of Interest Statement

The authors declare that the research was conducted in the absence of any commercial or financial relationships that could be construed as a potential conflict of interest. The handling editor declared a shared affiliation, though no other collaboration, with one of the authors (VA) and states that the process nevertheless met the standards of a fair and objective review.
